# A Novel Approach for Investigating Parkinson’s Disease Personality and Its Association With Clinical and Psychological Aspects

**DOI:** 10.3389/fpsyg.2019.02265

**Published:** 2019-10-11

**Authors:** Laura Carelli, Federica Solca, Silvia Torre, Jacopo Pasquini, Claudia Morelli, Rita Pezzati, Francesca Mancini, Andrea Ciammola, Vincenzo Silani, Barbara Poletti

**Affiliations:** ^1^Department of Neurology and Laboratory of Neuroscience, Italian Auxological Institute (IRCCS), Milan, Italy; ^2^Department of Pathophysiology and Transplantation, “Dino Ferrari” Center, University of Milan, Milan, Italy; ^3^Department of Business Economics, Health and Social Care, University of Applied Sciences and Arts of Southern Switzerland, Manno, Switzerland; ^4^Centro Terapia Cognitiva, Como, Italy

**Keywords:** Parkinson’s disease, personality, obsessive, quality of life, Personal Meaning Questionnaire

## Abstract

**Objective:**

A complex relationship between neuropsychiatric symptoms, personality traits and neurochemical changes in patients with Parkinson’s disease (PD) has been highlighted in the past several decades. In particular, a specific Parkinson personality with obsessive traits has been described. However, despite the great amount of anecdotal evidence, this aspect, together with its neurobiological, psychological and clinical correlates, are still not clearly defined. Therefore, we performed a case-control study in order to investigate the presence and rate of obsessive personality traits in PD patients within the theoretical framework of cognitive-constructivist model. Moreover, the relationship between PD personality and clinical, psychological and quality of life (QoL) aspects in PD were investigated.

**Methods:**

Fifty-one non-demented patients with probable or possible PD (not demented) were recruited at the inpatient-outpatient San Luca Hospital, IRCCS Istituto Auxologico Italiano. Control group was composed by forty-eight age- and education-matched healthy volunteers. Patients underwent a neurological investigation including Unified PD Rating Scale (UPDRS), Modified Hoehn and Yahr and Schwab and England staging scales. The following psychological questionnaires were administered to the overall sample: Personal Meaning Questionnaire (PMQ), State-Trait Anxiety Inventory-Form Y (STAI-Y), Beck Depression Inventory (BDI), Symptom Check List-90 (SCL-90), Short-Form Health Survey-36 (SF-36).

**Results:**

No significant differences in personality styles were observed in PD patients and controls, with a prevalence of phobic personal meaning organization (PMO) in both groups. However, PD patients showed more anxiety, depression and obsessive-compulsive (OC) symptoms than controls at the psychological questionnaires, as well as poorer QoL levels. The intensity of personality traits, and in particular for the obsessive personality style, were negatively associated with QoL and positively with disease severity. No significant relationships were observed between personality and other clinical aspects, such as side of onset and disease duration.

**Conclusion:**

Parkinson’s disease patients did not show a different personality profile according to the cognitive-constructivist model with respect to controls. However, in this population, a general enhancement in the tendency to codify experience by means of specific cognitive and emotional patterns was associated to disease progression and to a poorer QoL.

## Introduction

Parkinson’s disease (PD) is a chronic neurodegenerative disorder involving both motor and non-motor manifestations. In addition to cognitive impairment and sleep disorders, patients with PD often present with neuropsychiatric symptoms such as depression, anxiety, apathy and impulse control disorders (ICDs) as common non-motor symptoms. A complex relationship between neuropsychiatric symptoms and neurochemical changes (e.g., frontostriatal circuits and related dopamine functions) in PD has been highlighted in the past several decades (Porter, 2016). In particular, ICD and apathy respectively represent hyper- and hypo-dopaminergic symptoms and they are both influenced by the introduction of dopaminergic drugs (Sierra et al., 2015). Together with such aspects, a higher incidence of obsessive-compulsive (OC) symptoms has been recorded in PD, although with heterogeneous results (Maia et al., 2003; Harbishettar et al., 2005; Kummer et al., 2009; Sharp et al., 2015). The association between OC disorder and PD seems to be supported by the common involvement of frontostriatal circuits in these disorders and further suggested by an improvement of OC symptoms after subthalamic stimulation (Williams et al., 2016). Moreover, a relationship between OC symptoms and lateralization of motor symptoms in PD patients has been detected (Kummer and Teixeira, 2009b).

A high prevalence of OC personality disorder has also been observed in PD patients, both drug-naïve and under dopamine replacement therapy (Nicoletti et al., 2013, 2015). Therefore, it can be hypothesized that OC personality disorder can be an early manifestation of PD, partially independent from treatment.

A “parkinsonian personality” has been described since early reports in the past century by means of several case-report, case series, twin studies and case-control studies (Porter, 2016; Santangelo et al., 2017, 2018).

The presence of premorbid personality profile in PD patients has not been consistently supported by empirical evidence, while the study of personality in patients with established PD has led to more robust findings (Poletti and Bonuccelli, 2012; Santangelo et al., 2018). Most of the collected evidence arises from personality inventories developed according to the Cloninger’s psychobiological model, that proposed three temperamental dimensions based on three independent neurobiological systems: the Novelty Seeking (dopaminergic system); the Harm Avoidance (serotoninergic system); the Reward Dependence (noradrenergic system) (Cloninger et al., 1993). Within this frame of reference, it has been reported that PD patients are characterized by reduced “novelty seeking” and high “harm avoidance.” Personality in PD has also been assessed with tools developed according to the Big-Five Model (McCrae and Costa, 1997); results have shown higher level of neuroticism and lower levels of Openness and Extraversion as distinguishing characteristics of PD compared to healthy subjects (Santangelo et al., 2018). Some relationships have also been observed between PD patients’ personality and smoking habits, showing smoking as a significant mediator in the relationship between personality traits and PD (Sieurin et al., 2016) and revealing an association between smoking and novelty seeking (Menza, 2000).

Despite the use of heterogeneous assessment instruments and the varying qualities of the studies investigating OC symptoms and personality traits in PD, features of introversion, introspectiveness, inflexibility, industriousness, cautiousness, morality and an anancastic personality type have been consistently detected in such population.

These aspects converge in the description of an “obsessive organization” of personality proposed by Guidano within the cognitive-constructivist model within the field of psychotherapy. Guidano described four basic patterns of self-coherence, elaborated from the observation of characteristic cognitive styles and attachment patterns in some psychopathological conditions. Four “personal meaning organization styles” (PMO) have been described: depressive, phobic, eating disorder, and obsessive prone (Guidano, 1981, 1987). After successive elaborations of this theory, the organizations were finally described as “personality styles” which can be observed also in non-clinical populations (Arciero, 2002; Picardi et al., 2003; Arciero et al., 2004). Guidano focused on individual differences in the construction of a sense of self, and his model is typological rather than dimensional. In particular, individuals with an Obsessive PMO are characterized by a sense of self primarily based on conscious control of behavior and thinking, both of which are expected to match abstract principles; main represented themes are that of responsibility, anticipatory control, equity, order, certainty and coherence. The development by Picardi and colleagues (Picardi and Mannino, 2001; Picardi et al., 2003, 2004) of the Personal Meaning Questionnaire (PMQ), aimed at assessing the construct of PMO, provided new methods of personality investigation within the cognitive-constructivist paradigm. This instruments has been preliminarily used for the evaluation of personality characteristics of neurological populations (Poletti et al., 2017). Due to the categorical approach entailed in the PMQ, it seems useful to detect the presence of a specific and prevalent personality style and could improve the characterization of a parkinsonian personality. Moreover, in the validation study, PMQ cognitive-emotional organizations were compared to a set of criterion measures based on Cloninger’s and Big Five Models, showing a good external validity (Picardi et al., 2003, 2004). In the last decades, there has been increasing recognition of the importance to address quality of life (QoL) of PD patients, aside to managing the motor manifestations of the disorder. QoL in PD is influenced by psychological symptoms, which are partially related to dispositional attitudes or personality traits (Soh et al., 2011; van Uem et al., 2016; Kim et al., 2017; Pontone et al., 2017). Therefore, in addition to provide useful insights into the underlying neurochemical and biological changes along PD progression, the study of personality could help health professionals to improve the assessment process and address treatment strategies improving functional and emotional well-being in PD.

In the present case-control study, we aim to investigate the following aspects: the presence of higher rates, and higher prevalence, of obsessive personality traits in a sample of PD patients compared to age- and education-matched healthy controls; the possible association of obsessive personality traits with PD clinical factors (side of onset of motor symptoms, disease severity and duration, smoking habits); the relationship between personality traits and psychological symptoms commonly observed in PD such as depression, anxiety and OC symptoms; finally, the impact of personality traits on patients’ QoL.

## Materials and Methods

### Subjects

Fifty-one patients diagnosed with probable or possible PD according to Gelb et al. (1999) criteria were recruited at the inpatient-outpatient San Luca Hospital, IRCCS Istituto Auxologico Italiano. All but one patients were outpatients receiving ambulatory care from neurologists after hospital discharge. The only patients tested during the hospital recovery was evaluated soon after the admission. The mean age of patients was 68.20 years (SD = 9.19; range 47–81 years); mean education was 12.82 years (SD = 3.96; range 5–18 years). Marital and employment status were as follows: about 90% of patients was married, the remaining being divorced or single. About 65% were retired and 31% were still working; the remaining never worked. Forty-eight age- and education-matched healthy participants were recruited as control group among volunteers from local associations and researchers’ friends and relatives. The mean age of controls was 66.10 years (SD = 7.13; range 51–83 years); mean education was 13.00 years (SD = 3.45; range 5–18 years). About 90% of participants was married, with the remaining being widowed, divorced or single. For employment status, about half of them was retired, while 40% was actually working and the remaining has never worked.

Data were collected between March and December 2018.

Inclusion criteria for patients were: absence of dementia (according to clinical examination and Mini Mental State Examination – MMSE > 24/30); adequate educational level required for filling out the psychological questionnaires (≥5 years of education); if any, a stable psychiatric disorder (anxiety or depression) without relevant fluctuations at the moment of evaluation, as documented by our consultant neurologists at the last visit prior to the inclusion in the study.

Inclusion criteria for control participants were the absence of a history of neurological or psychiatric disorders and minimum educational level as for patients.

The study protocol was approved by the Ethics Committee of IRCCS Istituto Auxologico Italiano and all eligible subjects received verbal and written information about the study. All participants signed an informed consent, according to the Declaration of Helsinki.

### Clinical Examination

In PD patients motor severity and disease stage were assessed through the Unified PD Rating Scale (UPDRS; Fahn and Elton, 1987), the Modified Hoehn and Yahr (Goetz et al., 2004) and the Schwab and England (Schwab and England, 1969) scales.

Unified PD Rating Scale consists of four subscales: Section I (Mentation, behavior, and mood); Section II (Activities of daily living); Section III (Motor examination); Section IV (Complications of therapy). The total scores of the subscales are: Section I. 16 points; Section II. 52; Section III. 108; and Section IV. 23. The UPDRS total score ranges between 0 and 199 points.

The Modified Hoehn and Yahr describes seven stages of disease progression, from stage one, the earliest stage (unilateral involvement only), to stage five, the most severe (wheelchair bound or bedridden unless aided).

The Schwab and England Activities of Daily Living Scale rates patients’ level of disability from 0 to 100%: a score of 0% refers to bedridden patient and impaired vegetative functions (such as swallowing, bladder and bowel function); a score of 100% refers to a completely independent patient, able to manage its daily activities without slowness, difficulty, or impairment.

For each patient, the following aspects were also recorded: side of motor symptoms onset; disease duration (months); antiparkinsonian and psychotropic therapies; smoking habits prior and after disease onset.

### Psychological and Personality Assessment

Each subject underwent a battery of assessments investigating personality, psychological and QoL features: The PMQ, the State-Trait Anxiety Inventory-Form Y (STAI-Y), the Beck Depression Inventory (BDI), the Symptom Check List-90-R (SCL-90-R) and the Short-Form Health Survey-36 (SF-36). In the present study, we adopted the validated Italian version of each questionnaire. The described psychological and personality questionnaires have been adopted in a previous study aimed to evaluate personality features in other neurological populations (Poletti et al., 2017).

The PMQ consists of a self-report questionnaire (Picardi and Mannino, 2001; Picardi, 2003; Picardi et al., 2003) aimed to explore thoughts, feelings and behaviors as expected in the different PMOs. It is composed of 68 items to be answered according to the level of agreement/disagreement or to a 5-points Likert scale. Four 17-items subscales are present, each referring to a specific PMO: “phobic” (PP); “depressive” (DP); “psychogenic eating disorders” (EDP); “obsessive” (OP). PMQ Italian validation study showed the following psychometric properties: internal consistency – Cronbach’s alpha: 0.65–0.82; test-retest reliability – intra-class correlation: 0.58–0.84.

The STAI-Y (Spielberger, 1983; Pedrabissi and Santinello, 1989) is aimed at detecting and evaluating anxiety symptoms, concerning both state (STAI-Y1) and trait (STAI-Y2) anxiety components. It includes 40 questions, to be answered according to both intensity and frequency of symptoms, with scores ranging from 20 to 80. Internal consistency coefficients for the scale have ranged from 0.86 to 0.95; test-retest reliability coefficients have ranged from 0.65 to 0.75 over a 2-month interval (Spielberger, 1983).

The BDI (Beck et al., 1979; Centomo and Sanavio, Unpublished) evaluates depressive symptomatology, concerning somatic, emotional, cognitive and motivational components. The BDI consists of 21 items, concerning both cognitive-affective (BDI CA, items 0–13) and somatic-performance (BDI SP, items 14–21) symptoms of depression. Total score ranges from 0 to 63, with higher total scores indicating more severe depressive symptoms. According to BDI psychometric properties, the Italian validation showed a good internal consistency (Cronbach’s alpha = 0.82) and good test-retest reliability (0.74) (Centomo and Sanavio, Unpublished).

The SCL-90 inventory (Derogatis, 1994; Sarno et al., 2011) was employed in order to evaluate a broad range of psychopathological symptoms. This scale contains 90 items, highlighting mental disease according to 9 symptoms dimensions: somatization (SOM), OC, interpersonal sensitivity (IS), depression (DEP), anxiety (ANX), hostility (HOS), phobic anxiety (PHOB), paranoid ideation (PAR), and psychoticism (PSY). Evaluation of psychometric properties of the Italian version showed a good internal consistency for all subscales (values between 0.70 and 0.96) (Prunas et al., 2012).

A further questionnaire, the SF-36 (Ware and Sherbourne, 1992; Apolone et al., 1997), was also administered in order to detect health related QoL, consisting of a multidimensional tool made of 36 questions. Items refer to eight different health domains: physical functioning (PF), role limitations due to physical health (RP), bodily pain (BP), general health (GH), energy (EN), social functioning (SF), role limitations due to emotional problems (RE), and mental health (MH). The SF-36 has eight scaled scores; the scores are the weighted sums of the questions in each section. Scores range from 0 – 100, with lower scores indicating more severe disability. Moreover, the eight scales can be aggregated into two summary measures: the Physical Component Summary (PCS) for the physical dimension and the Mental Component Summary (MCS) for the mental dimension. The scoring algorithm for PCS and MCS were derived from an SPSS syntax for the Italian version of SF-36 developed by the Mario Negri Institute^1^. SF-36 psychometric assessment showed Cronbach’s alpha coefficients ranging from 0.77 to 0.93 and high internal consistency reliability among different subgroups with lower values in the GH scale, the young age groups and the more educated samples (Apolone and Mosconi, 1998).

A researcher neuropsychologist administered the cognitive screening tool for patients (MMSE). Data were collected with the supervision of the neuropsychologist who assisted the patients in the compilation of the self-report questionnaires.

### Statistical Analysis

Descriptive data are reported as means ± standard deviations for continuous variable and as absolute numbers for categorical variables. Normality of continuous variables was assessed with the Shapiro–Wilk test; the homogeneity of variances was assessed with Bartlett test. According to the test for normality of data, a normal distribution of PMQ subscores was observed, while psychological questionnaires subscores were almost totally not normally distributed. Thus, a mixed-design ANOVA model was used, with clinical condition (PD vs. control) as between-subjects variable and PMQ subscores as within-subjects variable with pairwise *t* test as *post hoc*. A Mann-Whitney U test was employed for comparing PD and control’s scores at the psychological questionnaires. In addition to total scores at each PMQ subscale (EDP, DP, PP, and OP), for each individual the presence of a prevalent PMO was calculated using the subscale where total value was at least 10% higher of the second most important subscale. A chi square test was employed in order to assess possible differences in prevalent PMO distribution among groups.

Correlational analyses between variables was carried out through a bivariate Pearson’s correlation analysis (*r*) if the assumptions of normality and homoscedasticity were verified; otherwise, a Spearman’s rank correlation analysis (*r*_s_) was employed.

The resulting *p*-values were corrected through a Benjamini–Hochberg procedure for false discovery rate. An α level of 0.05 was used for all hypothesis tests. All data analyses were performed using SPSS Statistics 24.

## Results

### Characteristics of Subjects

Descriptive statistics concerning demographical and psychological characteristics of PD patients and controls are summarized in Table 1.

**TABLE 1:** Demographic, cognitive and psychological characteristics of PD patients and controls.

	**PD patients (*N* = 51)**	**Healthy controls (*N* = 48)**			
	
	**Mean (SD)**	**Mean (SD)**	***t*/*U***	***p*-value**	**Cohen’s *d***
Age	68.20 (9.19)	66.10 (7.13)	1.26	0.21	0.25
Education	12.82 (3.96)	13.00 (3.45)	–0.24	0.81	0.05
MMSE correct score	27.89 (1.66)	28.89 (1.31)	−**2.89**	0.00	0.67
BDI total score	12.29 (9.16)	4.65 (3.86)	**542.50**	<0.001	1.09
BDI-CA	5.51 (5.41)	2.07 (2.31)	**666.50**	0.001	0.83
BDI-SP	6.76 (4.91)	2.73 (2.12)	**507.50**	<0.001	1.06
STAI-Y1	47.06 (9.77)	39.23 (5.97)	**582.00**	<0.001	0.97
STAI-Y2	49.67 (9.90)	42.92 (8.20)	**793.00**	0.003	0.74
SCL-90 SOM	11.00 (8.20)	6.00 (5.97)	**688.00**	<0.001	0.70
SCL-90 O-C	9.25 (7.08)	4.56 (4.54)	**686.50**	<0.001	0.79
SCL-90 IS	3.88 (3.56)	2.70 (2.99)	969.50	0.071	0.36
SCL-90 DEP	10.39 (7.60)	5.17 (4.23)	**650.00**	<0.001	0.85
SCL-90 ANX	6.71 (5.62)	3.15 (3.48)	**672.00**	<0.001	0.76
SCL-90 HOS	2.53 (2.93)	1.46 (1.75)	1008.00	0.120	0.44
SCL-90 PHOB	3.06 (4.55)	0.73 (1.28)	**767.50**	0.001	0.70
SCL-90 PAR	2.23 (2.26)	2.29 (3.08)	1077.50	0.294	0.02
SCL-90 PSY	3.61 (3.96)	1.69 (2.29)	**823.50**	0.004	0.59
SF-36 PF	67.70 (26.17)	86.35 (13.71)	**668.00**	<0.001	0.89
SF-36 RP	45.50 (37.35)	83.33 (27.93)	**525.00**	<0.001	1.15
SF-36 BP	51.36 (23.33)	75.04 (22.75)	**576.00**	<0.001	1.03
SF-36 GH	39.36 (16.82)	64.56 (19.09)	**366.00**	<0.001	1.40
SF-36 EN	50.10 (16.64)	67.91 (16.43)	**550.50**	<0.001	1.08
SF-36 SF	60.84 (20.49)	85.06 (16.42)	**450.50**	<0.001	1.30
SF-36 RE	56.66 (40.60)	84.06 (32.23)	**736.00**	<0.001	0.75
SF-36 MH	64.48 (20.53)	76.87 (15.17)	**743.00**	0.001	0.69
SF-36 PCS	49.15 (22.48)	76.49 (18.52)	408.00	<0.001	1.33
SF-36 MCS	59.38 (22.57)	76.11 (19.46)	659.00	<0.001	0.79
PMQ EDP	49.45 (8.80)	47.60 (9.72)	0.99	0.324	0.20
PMQ DP	42.25 (10.63)	39.94 (8.38)	1.19	0.233	0.24
PMQ PP	62.96 (9.65)	60.85 (8.08)	1.17	0.243	0.24
PMQ OP	59.23 (9.15)	58.69 (7.06)	0.33	0.741	0.07

*Bold numbers indicate statistical significance with *p* < 0.05. *t* = student’s *t* value; *U* = Mann–Whitney *U* test value; MMSE = mini mental state examination; BDI = Beck Depression Inventory; BDI-CA = cognitive-affective BDI; subscore; STAI-Y1 = State Anxiety Inventory-Form Y; STAI-Y2 = Trait Anxiety Inventory-Form Y; SF-36 = Short-Form Health Survey-36; SF-36 PF = physical functioning; SF-36 RP = role limitations due to physical health; SF-36 BP = bodily pain; SF-36 GH = general health; SF-36 EN = energy; SF-36 SF = social functioning; SF-36 RE = role limitations due to emotional problems; SF-36 MH = mental health; SCL-90 = Symptom Check List-90; SCL-90 SOM = somatization; SCL-90 OC = obsessive-compulsive; SCL-90 IS = interpersonal sensitivity; SCL-90 DEP = depression; SCL-90 ANX = anxiety; SCL-90 HOS = hostility; SCL-90 PHOB = phobic anxiety; SCL-90 PAR = paranoid ideation; SCL-90 PSY = psychoticism; PMQ = Personal Meaning Questionnaire; PMQ EDP = eating disorder personal meaning organization; PMQ DP = depressive personal meaning organization; PMQ PP = phobic personal meaning organization; PMQ OP = obsessive personal meaning organization.*

All patients were taking antiparkinsonian medication at the time of the evaluation. Of the 50 patients (for one patient this information was not available), eight were taking L-Dopa (LD) alone; eleven LD in combination with dopamine agonists (DAs); eight LD with Monoamine Oxidase inhibitors (MAO-I); ten LD with MAO-I and DA, three LD with MAO-I and Catechol-O-methyl transferase Inhibitors (COMT-I), one MAO-I only, one DA with MAO-I, two MAO-I in combination with LD and COMT-I, four LD in combination with DA, MAO-I and COMT-I, and two LD with other types of antiparkinsonian treatment. Moreover, 22 out of 51 patients (45%) were taking psychotropic drugs: nine were taking SSRI antidepressant; five were taking anxiolytic; seven were taking antiepileptic; three were taking antipsychotic (prescribed alone or in combination).

In PD patients, mean UPDRS total score was of 24.78 (SD = 18.84; range 4–96); mean disease duration was 7 years (SD = 5.5 years, range 1–24 years).

Information about PD patients smoking habits was available for 44 subjects: of these, 52% (*N* = 23) never smoked, 41% (*N* = 18) quit smoking, and 7% (*N* = 3) was still smoking at the time of the evaluation.

**FIGURE 1:**
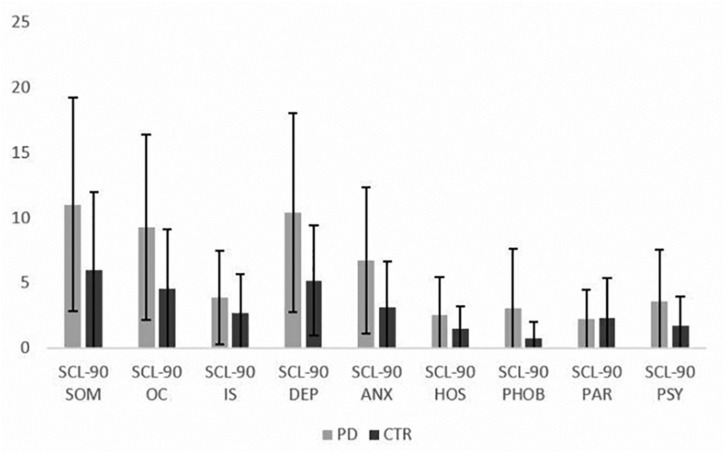
Mean scores at SCL-90 subscales in the PD patients and controls (mean ± SD). SCL-90 = Symptom Check List-90; SOM = somatization; OC = obsessive-compulsive; IS = interpersonal sensitivity; DEP = depression; ANX = anxiety; HOS = hostility; PHOB = phobic anxiety; PAR = paranoid ideation; PSY = psychoticism.

Patients presented with higher level of psychological symptoms than controls for several investigated domains (depression, anxiety, somatization, OC symptoms, psychoticism) (Table 1 and Figure 1). Moreover, QoL was lower in the patients compared to controls, with a significant reduction in all dimensions of SF-36 and in the two subcomponents concerning physical and mental QoL. These results and between groups comparisons at the psychological questionnaires are reported in Table 1.

### Personality Traits and PMO Prevalence

**FIGURE 2:**
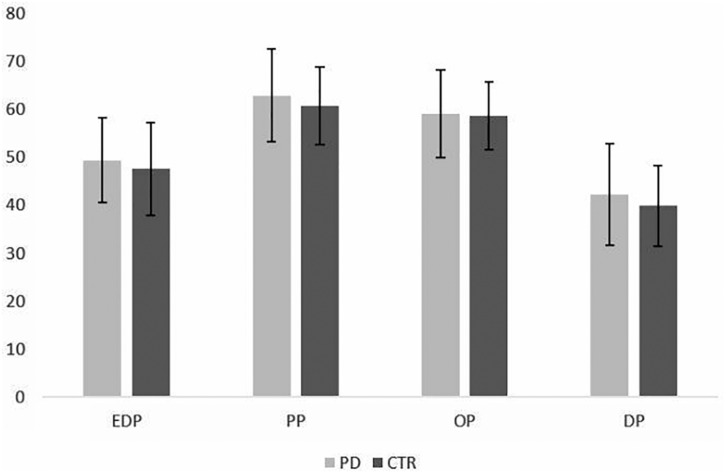
Distribution of PMQ mean scores in PD patients and control subjects (mean ± SD). EDP = eating disorder-prone PMO; DP = depression-prone PMO; PP = phobia-prone PMO; OP = obsessive-compulsive prone PMO.

The mixed ANOVA showed the presence of within groups differences in PMQ subscales scores (*p* < 0.001), while no between group differences were detected in the four PMOs mean values (*p* = 0.246). The pairwise *t*-test showed the presence of significant differences among each PMO subscale in both patients and controls with comparable distribution of higher mean values; in particular, the highest values were observed for PP, followed by OP, EDP, and DP subscales (see Table 1 and Figure 2). The differences among subscales were more significant for PD patients (*p* = 0.000) than for controls (*p* ranging between 0.055 and 0.000).

**FIGURE 3:**
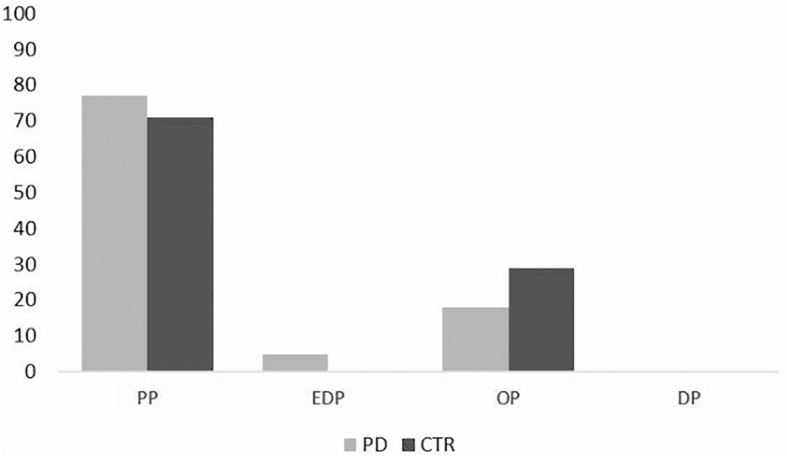
Prevalence of PMOs in PD patients and control subjects (%). EDP = eating disorder-prone PMO; DP = depression-prone PMO; PP = phobia-prone PMO; OP = obsessive-compulsive prone PMO.

Moreover, a total of 22 PD patients (43%) and 17 controls (35%) were classified as having a prevalent PMO (Figure 3). For PD patients, 77% (*N* = 17) showed a prevalent PP personality organization, 18% (*N* = 4) an OP and 5% (*N* = 1) an EP PMO. In controls, a majority of participants (71%, *N* = 12) showed a PP PMO and 29% (*N* = 5) an OP one. The Chi square showed no significant difference between patients and controls in prevalent PMOs distributions (*p* = 0.663).

In the PD group, significant correlations were observed between PMOs mean values and scores obtained at the psychological questionnaires. In particular, all PMQ subscales correlated with depression (BDI total score and cognitive-affective subscale, SCL-90-DEP; *p* ranging between <0.001 and 0.049; *r*_s_ values between 0.293 and 0.565), anxiety (STAI-Y2, SCL-90-phob, SCL-90-ANX; *p* ranging between <0.001 and 0.035; *r*_s_ between 0.315 and 0.486), SOM (SCL-90-SOM, *p* between 0.004 and 0.012; *r*_s_ values between 0.292 and 0.467), OC symptoms (SCL-90-OC, *p*-values between <0.001 and 0.016; *r*_s_ between 0.302 and 0.497), PAR (SCL-90-PAR, *p* between <0.001 and 0.021; *r*_s_ values between 0.395 and 0.517) and PSY (SCL-90-PSY, *p* between <0.001 and 0.010; *r*_s_ between 0.454 and 0.539).

In controls, fewer correlations were observed between PMOs mean values and psychological symptoms. In particular, only DP subscale correlated with depression (BDI total score – DP, *r*_s_ = 0.454, *p* = 0.024; SCL-90-DEP – DP, *r*_s_ = 0.391, *p* = 0.048); EDP and DP PMOs with trait anxiety at STAI-Y2, with *r*_s_ ranging between 0.314 and 0.479 and *p* = 0.024. Finally, EDP correlated with OC symptoms at SCL-90 (*r*_s_ = 0.441, *p* = 0.024).

### Clinical Correlates of Personality in PD

No correlation was observed between disease duration (months) and PMQ (EDP *r* = 0.063, *p* = 0.708; PP *r* = 0.144, *p* = 0.387; OP *r* = 0.141, *p* = 0.346; DP *r* = −0.059, *p* = 0.705).

No significant differences in PMQ subscales were found between patients with right or left side onset of PD: PP (*t* = 0.259, *p* = 0.797), EDP (*t* = 0.745, *p* = 0.460), OP (*t* = −0.084, *p* = 0.933), and DP (*t* = 0.238, *p* = 0.813).

Correlational analysis between obsessive personality traits and disease severity showed a positive association between OP mean values and UPDRS total score (*r*_s_ = 0.416, *p* = 0.007), as well as UPDRS III-Motor Examination (*r*_s_ = 0.497, *p* = 0.004) subscore. DP and PP PMOs were associated with UPDRS total score (*r*_s_ = 0.373, *p* = 0.019; *r*_s_ = 0.400, *p* = 0.016 respectively) and with UPDRS III subscore (*r*_s_ = 0.414, *p* = 0.010; *r* = 0.445, *p* = 0.010, respectively). UPDRS II subscore was only associated to DP one (*r*_s_ = 0.351, *p* = 0.026).

A negative correlation was also observed between Schwab and England clinical scale total score and PMQ mean values for OP (*r*_s_ = −0.383, *p* = 0.019) and DP (*r*_s_ = −0.327, *p* = 0.032) PMOs. Conversely, the Modified Hoehn and Yahr staging scale score did not correlate with OP mean score (*r*_s_ = 0.086, *p* = 0.546), nor with the other PMQ subscales (*p* > 0.05).

Finally, we investigated the possible association of smoking habits with a specific PMO. This analysis was limited to the “former smoker” and “never smoker” categories, due to the paucity of subjects included in the “current smoker” group (*N* = 3). Comparison between the two groups showed the absence of significant differences between “never smoker” and “former smoker” for all PMOs: PP (*t* = 0.612, *p* = 0.544), EDP (*t* = −0.002, *p* = 0.999), OP (*t* = −0.270, *p* = 0.788) and DP (*t* = −0.520, *p* = 0.606) mean values.

### Relationships Between Personality Traits and Quality of Life

In the PD group we analyzed the relationship between OP PMO and QoL. We fund significant negative associations between OP PMO scores and SF-36 dimensions of general health (SF-36-GH, *r*_s_ = −0.309, *p* = 0.051) and role limitations due to emotional problems (SF-36-RE, *r*_s_ = −0.500; *p* < 0.001). Mild correlations were also observed between other PMOs and QoL, in particular concerning general health (PP *r*_s_ = −0.344, *p* = 0.036), role limitations due to emotional problems (DP *r*_s_ = −0.337, *p* = 0.032), mental health (EDP *r*_s_ = −0.339, *p* = 0.045; DP *r*_s_ = −0.366, *p* = 0.020) and PF (DP *r*_s_ = −0.334, *p* = 0.032) dimensions. Conversely, in the control group no correlation was observed between QoL and PMOs subscores. When considering SF-36 physical (PCS) and mental (MCS) subcomponents, mild to moderate correlations were observed in PD group between MCS and all PMOs (*r*_s_ between −0.365 and −0.450, *p* between 0.004 and 0.034) with the highest associations detected for obsessive PMO. In control participants, no correlation was observed between PMOs and PCS values.

## Discussion

The presence of a prevalent personality organization in PD patients, with respect to healthy controls, has not been confirmed in our study. Moreover, the hypotheses of higher prevalence and rates of obsessive personality traits in PD patients is not supported by our data. Conversely, both patients and controls scored higher in the phobic PMO subscale. The latter finding, in the cognitive-constructivist model, suggests the presence of alexitimic traits typical of phobic organization, consisting of a difficulty to identify emotional experiences and to relate emotional states to somatic perturbations. Such results are in accordance with Guidano’s considerations (Guidano, 1987) about distribution of PMO in the general population and with previous studies employing PMQ in other neurological conditions (Poletti et al., 2017; Possa et al., 2017). Moreover, these data are also in accordance with previous studies, highlighting an association between premorbid anxious personality and risk of developing PD (Bower et al., 2010). Conversely, our findings do not support anecdotal descriptions, and previous studies showing differences between patients and control subjects by means of assessment tools referring to the Big Five and Psychobiological Models (Poletti and Bonuccelli, 2012; Santangelo et al., 2017, 2018). However, the employed questionnaires (i.e., the NEO Personality Inventory, the Eysenck Personality Inventory, the Tridimensional Personality Questionnaire) are not designed to specifically evaluate personality styles, but rather to measure personality factors that contribute to different profiles. Differently, the PMQ describes four specific personality organizations, as specific rules for organizing immediate experience in order to maintain internal consistency and manage perturbations arising from the environment. Therefore, our results do not exclude the possible presence of specific personality traits or temperamental aspects in PD patients, premorbid or concurrent to disease onset; rather, they suggest that such traits, if present, do not contribute to define a specific personality style when measured with PMQ within the Guidano’s model. The combination of PMQ with other traditional personality inventories could help to better define this aspect in future studies.

Despite the absence of prevalent obsessive personality traits, our PD patients showed a significant higher rate of obsessive, anxiety and depressive symptoms, with respect to controls. These data are in accordance with current knowledge about PD patients showing a significant rate of neuropsychiatric symptoms in this population (Maia et al., 2003; Kummer and Teixeira, 2009a). In our sample, these symptoms showed a positive correlation with all PMOs in the patients’ group and, to a lesser extent, in controls; these findings suggest that the stiffening of PMOs’ characteristics, rather than the prevalence of a specific PMO, leads to a greater risk of developing psychological symptoms in PD patients.

In regard to clinical correlates of PD personality, we detected a significant correlation between PMQ subscores (in particular OP, DP, and PP) and disease severity (UPDRS), as well as global disability level (Schwab and England staging scale). These findings could suggest a general strengthening of all cognitive and emotional patterns as the disease progresses and disability increases, without any differences between PMOs. Conversely, no association was found between PMQ subscores and disease onset, and between symptom lateralization and personality traits. Most of current literature about PD has not found significant associations between personality traits, as well as OC symptoms, and clinical measures such as disease severity and duration (Maia et al., 2003; Harbishettar et al., 2005; Kummer and Teixeira, 2009b; Santangelo et al., 2017). Instead, a certain association between symptom lateralization and personality aspects have been found (Maia et al., 2003; Piacentini et al., 2011; Santangelo et al., 2017). Our contrasting results could be due to the different frame of reference for the study of personality and to the different assessment tools employed.

In the present study, we also aimed to investigate the relationship between smoking habits and personality aspects. Our findings did not show any difference between “never smoker” and “former smoker” patients on PMQ subscores. Considerations about “current smoker” patients have not been possible due to the paucity of patients in this category, in accordance with literature showing smoking as a protective factor for PD. It is possible that more information about smoking habits, such as number of cigarettes/day and years of smoking, and the recruitment of a larger sample of “current smoker” patients could provide more conclusive findings about this topic.

In regard to QoL, a specific effect of PD patients’ obsessive personality traits was observed in our study: the obsessive PMO was significantly and negatively correlated to QoL, suggesting a strong impact of such personality style in determining both poor general health and role limitations due to emotional problems. Therefore, in PD, the strengthening of specific personality traits and cognitive-emotional experiences substantially reduce the adjustment capacity and coping abilities necessary to adapt to such a progressive disease. Therefore, it seems that PD does not modify the prevalence of different PMOs, but it could change the relationship between personality and QoL.

A number of limitations in our study should be addressed. First, patients’ recruitment did not consider the inclusion of recently diagnosed, drug-naïve patients; instead, all our patients were taking antiparkinsonian therapy. Thus, the effect of dopaminergic treatment on personality features could not be controlled, as well as the possible role of psychotropic drugs; such possible moderating aspects should be controlled in further analysis and investigations of PD personality. Then, in the patients’ selection process we excluded patients with overt dementia, and this could limit the generalization of our results to other PD populations with more severe cognitive impairment. However, by doing so, we assured that personality trait changes (and other psychological aspects) were not due to or influenced by a dementing process. The administration of self-report psychological questionnaires such as PMQ requires a certain degree of cognitive integrity and the absence of obvious anosognosia and anosodiaphoria; therefore, this limitation concerning sample selection could be overcome or compensated only by using other kind of measures such as informant-report ones. Moreover, the absence of a nosographic diagnosis concerning psychopatological and personality dimensions, not having included other more traditional personality assessment tools and the adoption of a cross-sectional study may limit the explanatory power of our results from a clinical point of view. Finally, the detection of higher phobic PMO across different neurological conditions (Poletti et al., 2017; Possa et al., 2017) could possibly underlie some methodological bias that should be addressed in order to improve PMQ validity and sensitivity.

As a consequence, our results cannot be considered conclusive in regard to the assessment of personality style in PD patients within the frame of cognitive-constructivist PMOs. In spite of these limitations, the findings reported in the present study represent the first contribution toward the understanding of the personality profiles in PD patients according to the Guidano’s cognitive-constructivist model.

A timely evaluation of personality in PD could help clinicians to identify those patients at higher risk of developing psychological disorders (anxiety, depression, OC symptoms), behavioral alterations and poorer QoL as a consequence of disease impact and progression.

We suggest that adequately assessing these profiles in clinical practice and neuro-rehabilitation settings may be relevant to plan pharmacological and/or psychological treatment tailored on each patient’s characteristics.

## Data Availability Statement

Datasets are available on request. The raw data supporting the conclusions of this manuscript will be made available by the authors, without undue reservation, to any qualified researcher.

## Ethics Statement

This study was carried out in accordance with 2016/679 EU Regulation, with written informed consent from all subjects. All subjects gave written informed consent in accordance with the Declaration of Helsinki. The protocol was approved by the Ethics Committee of the IRCCS Istituto Auxologico Italiano.

## Author Contributions

LC was responsible for the study design. LC and BP wrote the manuscript. FS was responsible for the ALS patients assessment. ST was responsible for the healthy controls assessment. AC, JP, CM, and FM were responsible for the patients’ neurological examination. RP contributed in designing the study. VS critically revised the manuscript. All authors approved the final version of the manuscript.

## Conflict of Interest

The authors declare that the research was conducted in the absence of any commercial or financial relationships that could be construed as a potential conflict of interest.

## Footnotes

1 http://lsi.marionegri.it/qdv/downloads/SF36%20SPSS.SPS
